# Effect of Host Structure on Optical Freedericksz Transition in Dye-Doped Liquid Crystals

**DOI:** 10.3390/ma15124125

**Published:** 2022-06-10

**Authors:** Junki Yokota, Kohsuke Matsumoto, Koji Usui, Shoichi Kubo, Atsushi Shishido

**Affiliations:** 1 Laboratory for Chemistry and Life Science, Institute of Innovative Research, Tokyo Institute of Technology, R1-12, 4259 Nagatsuta, Midori-ku, Yokohama 226-8503, Japan; jyokota-st@polymer.res.titech.ac.jp (J.Y.); kmatsumoto-st@polymer.res.titech.ac.jp (K.M.); kusui-st@polymer.res.titech.ac.jp (K.U.); kubo@res.titech.ac.jp (S.K.); 2 Department of Chemical Science and Engineering, School of Materials and Chemical Technology, Tokyo Institute of Technology, 2-12-1 Ookayama, Meguro-ku, Tokyo 152-8552, Japan

**Keywords:** optical Freedericksz transition, dye-doped liquid crystal, molecular reorientation

## Abstract

The optical Freedericksz transition (OFT) can reversibly control the molecular orientation of liquid crystals (LCs) only by light irradiation, leading to the development of all-optical devices, such as smart windows. In particular, oligothiophene-doped LCs show the highly sensitive OFT due to the interaction between dyes and an optical-electric field. However, the sensitivity is still low for the application to optical devices. It is necessary to understand the factors in LCs affecting the OFT behavior to reduce the sensitivity. In this study, we investigated the effect of the host LC structure on the OFT in oligothiophene-doped LCs. The threshold light intensity for the OFT in trifluorinated LCs was 42% lower than that in LCs without fluorine substituents. This result contributes to the material design for the low-threshold optical devices utilizing the OFT of dye-doped LCs.

## 1. Introduction

The Freedericksz transition is a phenomenon to reversibly change the molecular orientation of liquid crystals (LCs) by external stimuli, such as an electric, magnetic, and optical-electric field above a certain intensity. This phenomenon is applicable to functional optical devices, such as displays and smart windows [1,2,3]. In particular, the optical Freedericksz transition (OFT) induced only by an optical-electric field has attracted much attention due to the potential for the development of energy-saving devices driven by sunlight. However, the OFT still requires high light intensity above 10^2^ W/cm^2^ [4,5,6,7,8]. Therefore, enhancing the sensitivity of OFT, that is, the sensitivity of the molecular reorientation of LC is essential for the application to all-optical devices.

One approach to improve the sensitivity of the molecular reorientation is doping the LCs with small amounts of certain dichroic organic dyes. This approach was first reported by Jánossy et al., where the sensitivity of the molecular reorientation of LCs was enhanced by the addition of anthraquinone dyes, resulting in inducing the OFT at a ten-times lower light intensity than LCs without dyes [9,10,11]. The rod-like dyes excited by the irradiation with a linearly polarized laser beam are aligned parallel to the incident polarization direction due to the interaction with the optical-electric field. Consequently, LC molecules are also reoriented along the same direction as the dye molecules due to the cooperative interaction.

Instead of the anthraquinone dyes, Zhang et al. reported the usage of oligothiophene dyes to enhance the sensitivity of the LC molecular reorientation [12]. To further decrease the light intensity required for the OFT in oligothiophene-doped LCs, various approaches focusing on the incident light, alignment processing, and materials have been proposed [13,14,15,16,17,18,19]. Recently, we focused on the material design and achieved the effective molecular reorientation by doping oligothiophene dyes with ester moieties [17,18]. In addition, we found that incorporating a polymer into dye-doped LCs caused the enhancement and stabilization of the molecular reorientation compared to conventional low-molecular-weight LCs [19,20]. However, the effect of host LCs, which determines the physical properties of the LC systems, on the OFT remained unexplored in oligothiophene dye-doped LCs. Investigation of the effect of host LCs gives an insight into the material design for enhancing the sensitivity of the OFT.

In this work, we explored the OFT behavior in oligothiophene dye-doped LCs using fluorinated host LCs in terms of the effect of the electron withdrawing group. The OFT was measured by observing the diffraction rings caused by the molecular reorientation of LCs. The light intensity at which the molecular reorientation occurred depended on the structure of the host LCs. The dye-doped LCs using host LCs with three fluorine substituents induced the OFT at a lower light intensity than previously reported dye-doped LC. Furthermore, the dye-doped LCs containing host LCs with two fluorine substituents required a higher light intensity than those with three fluorine substituents. We revealed that the number of fluorine substituents of host LCs greatly affected the sensitivity of the OFT. This result can provide important knowledge for designing highly sensitive dye-doped LCs.

## 2. Materials and Methods

### 2.1. Sample Preparation

**Figure 1:**
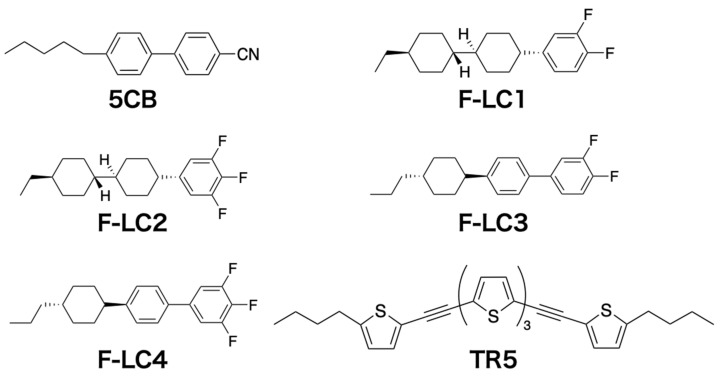
Chemical structures of the compounds used in this study.

Figure 1 shows the chemical structures of the compounds used in this study. As host LCs, we used 4-cyano-4′-pentyl biphenyl (5CB) obtained from Merck Ltd., Tokyo, Japan, and four types of fluorinated LCs (F-LCx, x = 1–4) obtained from Tokyo Chemical Industry Co. Ltd., Tokyo, Japan. F-LCx are as follows: *trans*-4-(3,4-difluorophenyl)-*trans*-4′-ethylbicyclohexane (F-LC1); *trans*,*trans*-4′-ethyl-4-(3,4,5-trifluorophenyl)bicyclohexyl (F-LC2); 3,4-difluoro-4′-(*trans*-4-propylcyclohexyl)biphenyl (F-LC3); and 3,4,5-trifluoro-4′-(*trans*-4-propylcyclohexyl)biphenyl (F-LC4). A dichroic oligothiophene dye molecule, 5,5′-bis-(5-butyl-2-thienylethynyl)-2,2′:5′,2”-terthiophene (TR5), was synthesized as reported previously [12].

Pure 5CB or mixtures of 5CB and F-LCx (5CB:F-LCx = 75:25, molar ratio) were mixed with a TR5 solution (0.01 mol/L in tetrahydrofuran (THF)) in brown vials, diluted in THF, and stirred for 1 h. After stirring, the solvent was completely removed under vacuum. The TR5 concentration was adjusted for each host mixture to show the same absorbance. The details are described in the “Result & Discussion” section.

**Figure 2:**
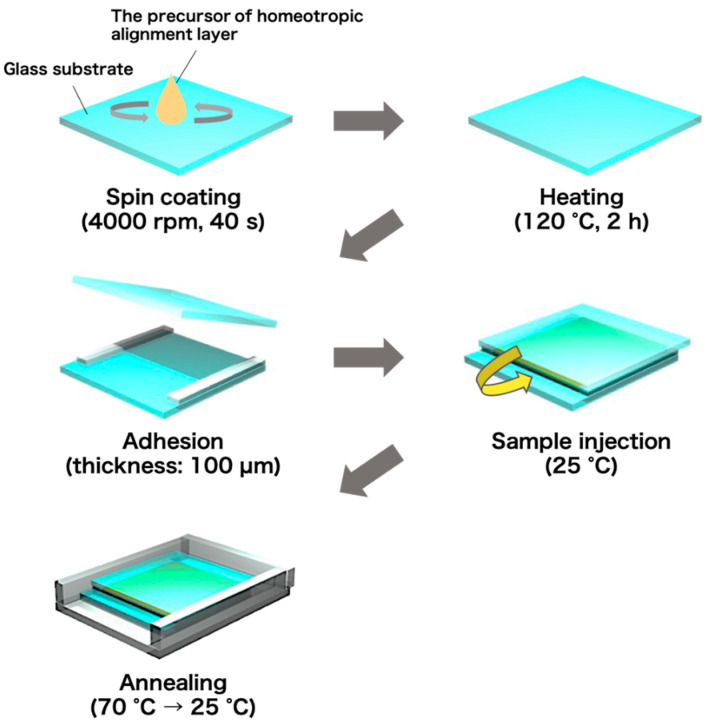
Preparation procedure for homeotropic-aligned oligothiophene-doped LC cells.

Glass cells with homeotropic alignment layers were fabricated according to the procedure shown in Figure 2. Glass substrates (25 mm × 25 mm) ultrasonicated in 2-propanol were treated with an ultraviolet (UV)-ozone cleaner (NL-UV42, Nippon Laser & Electronics Lab Co. Ltd., Nagoya, Japan) for 10 min to make the glass surface hydrophilic. A polyimide solution (SUNEVER, Nissan Chemical Co., Ltd., Tokyo, Japan) was spin-coated on the glass substrates at 4000 rpm for 40 s with a spin-coater (MS-A100, MIKASA, Co., Ltd., Tokyo, Japan), and heated at 120 °C for 2 h to obtain surface-treated glass substrates, which aligns LC molecules homeotropically (out-of-plane direction). Two surface-treated substrates were bonded with 100-μm-thick polyimide tapes to manufacture a glass cell. The thickness of the glass cell was measured using a micrometer (MDC-25MX, Mitutoyo Co., Kanagawa, Japan).

The dye-doped host LCs were injected into glass cells by capillary action. The glass cells were heated up to 70 °C to remove the disturbance of the molecular orientation by injection and then cooled down to room temperature at a cooling rate of 2 °C/min. We defined the five prepared cells depending on the compounds as follows: TR5/5CB; TR5/(5CB F-LC1); TR5/(5CB F-LC2); TR5/(5CB F-LC3); and TR5/(5CB F-LC4).

### 2.2. Evaluation of Initial Molecular Orientation

The initial molecular orientation in the samples was evaluated by conoscopic polarized optical microscopy (POM) and polarized ultraviolet (UV)–visible (vis) absorption spectroscopy. The conoscopic micrographs were obtained using a polarized optical microscope (BX53-P, Olympus Corp., Tokyo, Japan) equipped with an interference filter at 550 nm. The absorption spectra were measured with a UV–vis spectrophotometer (V-750, JASCO Corp., Tokyo, Japan) equipped with a rotatable film holder. Absorbances parallel and perpendicular to the direction of the sample injection were defined as *A*_∥_ and *A*_⊥_, respectively.

### 2.3. Self-Diffraction Ring Measurement

**Figure 3:**
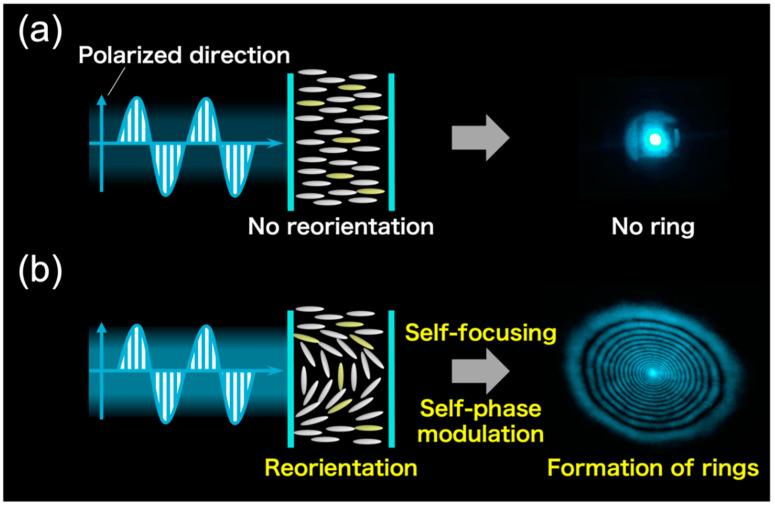
Principle of self-diffraction ring formation: (**a**) below and (**b**) above the threshold light intensity.

Photoinduced molecular reorientation was analyzed from self-diffraction rings. The diffraction ring is formed by the refractive index change of molecular reorientation caused by light irradiation above certain intensity. Irradiation of a homeotropic-aligned cell with a low-intensity Gaussian beam does not cause molecular reorientation (Figure 3a). When the light intensity exceeds the threshold of the molecular reorientation, the diffraction ring occurs due to self-focusing and self-phase modulation (Figure 3b) [8]. The number of rings (*N*) depends on the photoinduced refractive index change (∆*n*’) of the samples, given by

*N* = |∆*n*′|*Lλ^−^*^1^ (1)

where *L* and *λ* denote the cell thickness and the wavelength of the laser beam, respectively [21]. ∆*n*′ is the change in the refractive index of the sample. Rod-shaped LC molecules have an anisotropic refractive index in the short and long axes of the molecule. When photoinduced molecular reorientation from perpendicular to parallel to the polarization direction occurs, the refractive index becomes large, resulting in an increase in the number of rings.

**Figure 4:**
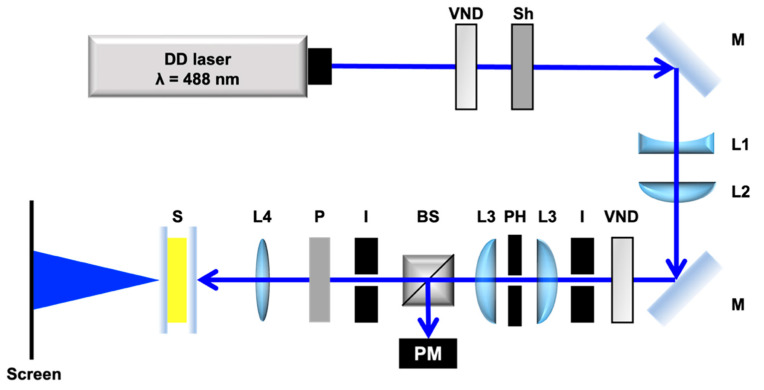
Optical setup for self-diffraction ring measurement. VND, variable neutral density filter; Sh, shutter; M, mirror; L1, plane concave lens (*f* = −80 mm); L2, plane convex lens (*f* = 150 mm); I, iris; L3, plane convex lens (*f* = 70 mm); PH, pinhole; BS, beam splitter; PM, power meter; P, polarizer; L4, biconvex lens (*f* = 150 mm); S, sample.

The optical setup for self-diffraction measurement is shown in Figure 4. We used a linearly polarized direct diode (DD) laser beam (EXLSR-488C-200-CDRH, Spectra-Physics, Inc., Tokyo, Japan) with a wavelength of 488 nm where TR5 has an absorption band. The beam diameter was expanded from 700 µm to 1.7 mm via lenses L1 and L2. Spatial filtering of the laser beam was performed with a pinhole (*φ* = 50 μm) and lenses L3. The laser beam was incident on the sample cell placed at the focal point of L4. The beam diameter at the focal point was 54 μm. The light intensity was controlled using a variable neutral density filter. The light intensity at the irradiation spot was defined as

*I* = *I*_0_π*w*^2^ (2)

where *w* and *I*_0_ are the beam waist and incident light power, respectively. *I*_0_ was measured using a power meter and a beam splitter with a definite ratio (Transmittance:Reflectance = 1:1). Transmitted light was projected onto a screen as self-diffraction rings. As the light intensity increases, the molecular reorientation is further induced, resulting in the increase in the number of rings. We counted the self-diffraction rings manually to evaluate the molecular reorientation of LCs in the cell. The threshold intensity of the molecular reorientation was defined as the light intensity at which the first ring appears. The first ring was monitored with a beam profiler (BGP-USB-SP620, Ophir-Spiricon LLC., North Logan, UT, USA).

### 2.4. Elastic Constant Measurement

A Frank elastic constant of the samples was measured with an elastic constant measurement system (EC-1, TOYO Corp., Tokyo, Japan). Glass cells coated with indium-tin-oxide (ITO) electrodes (KSRP-25/B111P1NSS) required for elastic constant measurement were obtained from EHC Co., Ltd., Tokyo, Japan. The glass substrates (25 mm × 20 mm) with the electrode area (10 mm × 10 mm) were treated with a homogeneous alignment layer (in-plane direction). The cell gap was 25 μm. Each sample was injected into the glass cells by capillary action. The glass cells were heated up to 70 °C and then cooled down to room temperature at a cooling rate of 2 °C/min.

## 3. Results and Discussion

**Figure 5:**
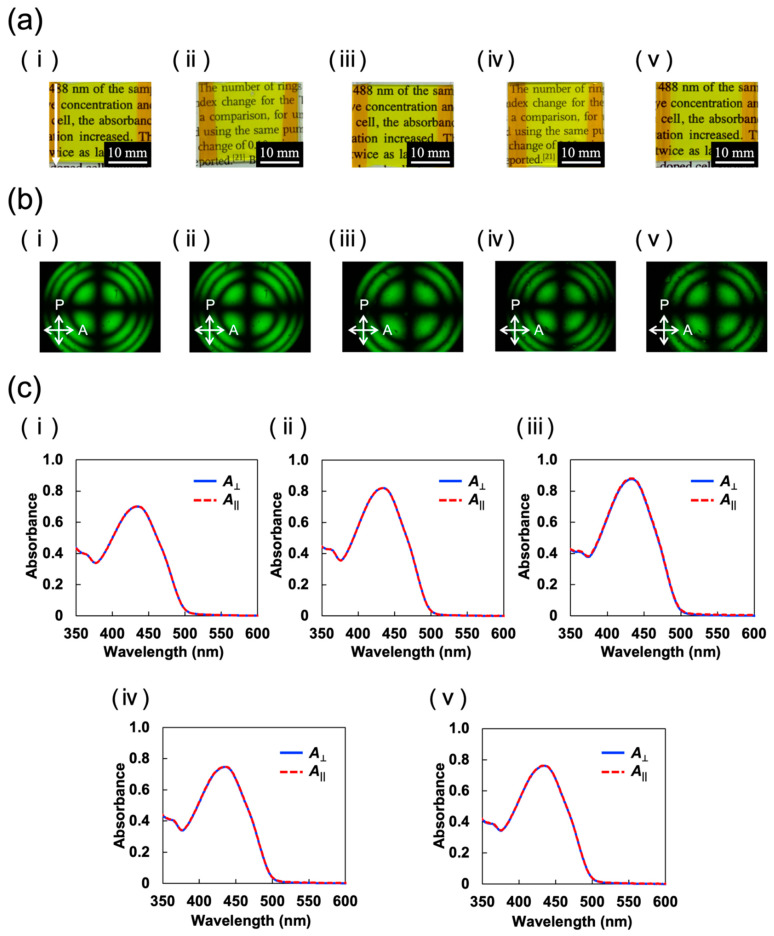
(**a**) Photographs, (**b**) conoscopic POM images, and (**c**) polarized UV-vis absorption spectra of prepared samples: (**i**) TR5/5CB, (**ii**) TR5/(5CB F-LC1), (**iii**) TR5/(5CB F-LC2), (**iv**) TR5/(5CB F-LC3), and (**v**) TR5/(5CB F-LC4). Blue solid and red dash lines in (**c**) denote the absorption perpendicular (*A*_⊥_) and parallel (*A*_∥_) to the direction of sample injection, respectively.

The prepared samples exhibited an optically transparent yellow color due to the absorption of TR5 (Figure 5a). Conoscopic micrographs of the samples showed a clear isogyre (Figure 5b). This means that the optic axes of LC molecules in the samples are perpendicular to the glass substrate. Furthermore, the UV–vis absorption spectra perpendicular (*A*_⊥_) and parallel (*A*_∥_) to the direction of the sample injection were identical in the wavelength range from 350 to 600 nm (Figure 5c). TR5 is a dichroic dye with anisotropic absorption in the short and long axes of the molecule. Therefore, this result demonstrates that the long axis of the TR5 molecules are perpendicular to both incident orthogonal polarizations and that both host LCs and TR5 in each cell are aligned uniformly and homeotropically.

Table 1 shows the concentration of TR5 in each sample and the absorbance at a wavelength of 488 nm obtained from absorbance spectra of an average of the *A*_⊥_ and *A*_∥_. An increase in the absorbance at 488 nm decreases the threshold intensity [17]. Therefore, the concentration of TR5 in each sample was adjusted so that the absorbance at 488 nm was almost the same for each sample: the absorbance of TR5/5CB, TR5/(5CB F-LC1), TR5/(5CB F-LC2), TR5/(5CB F-LC3), and TR5/(5CB F-LC4) were 0.155, 0.155, 0.162, 0.161, and 0.158, respectively.

**Figure 6:**
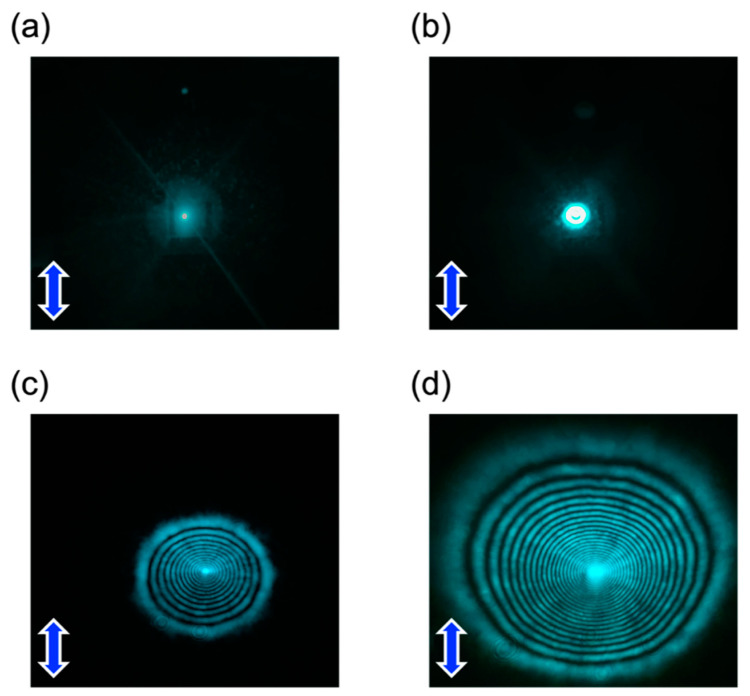
Diffraction ring images of TR5/5CB with the incident light intensity of (**a**) 15, (**b**) 20, (**c**) 27, and (**d**) 55 W/cm^2^. Blue arrows indicate the polarization direction of the incident light.

The OFT was investigated from the diffraction rings. Figure 6 displays the transmitted light from the TR5/5CB cell. The incidence of a laser beam with a low light intensity did not cause the molecular reorientation, thus no diffraction ring appeared (Figure 6a). When the intensity exceeded 20.1 W/cm^2^, the first diffraction ring appeared on the screen (Figure 6b). Furthermore, the number of the rings increased as the light intensity increased up to 54.6 W/cm^2^ (Figure 6b–d), and became constant.

**Figure 7:**
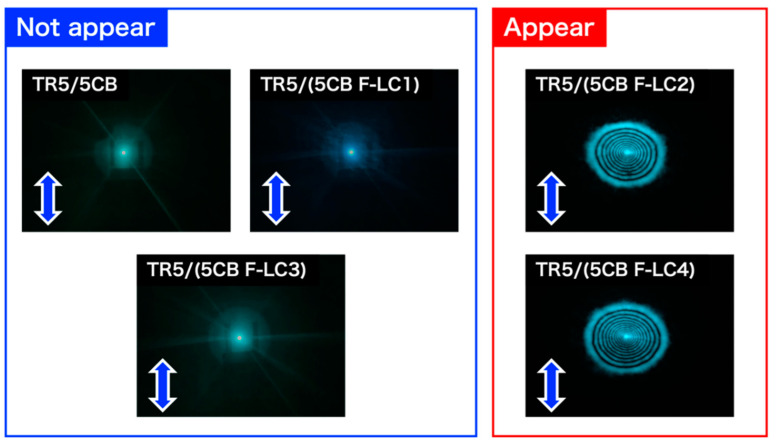
Typical diffraction patterns of all samples induced by a multi-mode laser beam formed at 18 W/cm^2^. Blue arrows indicate the polarization direction of the incident light.

The threshold light intensity inducing the diffraction rings depended on the host LCs. Figure 7 shows the diffraction patterns of each sample formed on a screen at a light intensity of 18 W/cm^2^. Diffraction rings appeared in TR5/(5CB F-LC2) and TR5/(5CB F-LC4); in contrast, they did not appear in TR5/5CB, TR5/(5CB F-LC1), and TR5/(5CB F-LC3). This indicates that the sensitivity of light-induced molecular reorientation is different in each sample.

**Figure 8:**
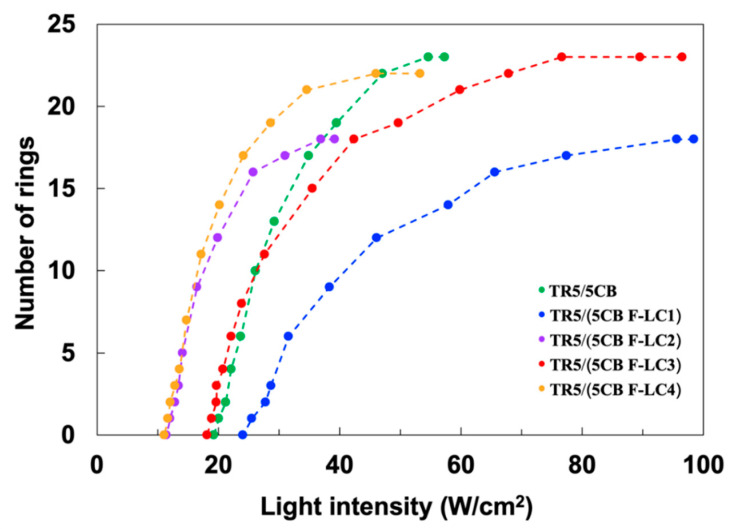
Number of diffraction rings of TR5/5CB, TR5/(5CB F-LC1), TR5/(5CB F-LC2), TR5/(5CB F-LC3),and TR5/(5CB F-LC4) as a function of light intensity.

Figure 8 shows the number of diffraction rings as a function of light intensity, and Table 2 summarizes the threshold intensity, the maximum number of rings, and *K*_33_ elastic constant of the samples. The threshold intensities of TR5/(5CB F-LC2) and TR5/(5CB F-LC4), which contain the LCs with three fluorine substituents, were 12.0 W/cm^2^ and 11.7 W/cm^2^, respectively. These are 42% lower than that of TR5/5CB (20.1 W/cm^2^). Furthermore, TR5/(5CB F-LC1) and TR5/(5CB F-LC3), which contain the LCs with two fluorine substituents, exhibited higher threshold intensities than TR5/(5CB F-LC2) and TR5/(5CB F-LC4). This threshold intensity difference indicates that the sensitivity of light-induced molecular reorientation is affected by the number of fluorine substituents in the host LCs. Furthermore, the maximum number of rings of TR5/5CB, TR5/(5CB F-LC1), TR5/(5CB F-LC2), TR5/(5CB F-LC3), and TR5/(5CB F-LC4) were 23, 18, 18, 23, and 22, respectively. TR5/5CB, TR5/(5CB F-LC3), and TR5/(5CB F-LC4), which showed a larger number of diffraction rings, have biphenyl mesogenic structures.

We discuss the OFT of each sample. The number of rings is governed by a sample cell thickness, wavelength of the incident light, and refractive index change, as shown in Equation (1). In this experiment, the cell thickness (100 µm) and the wavelength (488 nm) are the same for all samples. Therefore, the difference in the maximum number of rings is derived from the maximum refractive index change $∆n_{\max}^{\prime }$ of the sample. The value of $∆n_{\max}^{\prime }$ of 5CB, F-LC1, F-LC2, F-LC3, and F-LC4 was estimated to be 0.11, 0.09, 0.09, 0.11, and 0.11, respectively. 5CB, F-LC3, and F-LC4 with a biphenyl structure have longer conjugation lengths than F-LC1 and F-LC2 with a phenyl ring. The biphenyl structure tends to have a larger birefringence because of the anisotropic delocalization of electrons. This may lead to the difference in the maximum refractive index change of the sample even with the similar molecular reorientation.

The difference in the threshold intensity, which reflects the sensitivity of molecular reorientation, is derived from the dielectric anisotropy of each sample. According to previous work, the threshold intensity is determined by a balance of four torques: optical-electric torque, elastic torque, dye torque, and surface anchoring [15]. The increase in the optical-electric torque promotes the molecular reorientation. The optical-electric torque correlates with the dielectric anisotropy of LCs [10]. In general, the dielectric anisotropy of LCs becomes positive and increases by fluorine substitution [22]. In fact, the three fluorine substituents at the terminal position of a mesogen core enhance the dielectric anisotropy along the long axis of the LC molecule [22,23]. In our materials, the dielectric anisotropy of F-LC2 and F-LC4 modified with three fluorine substituents is larger than that of F-LC1 and F-LC3 modified with two fluorine substituents, which could increase the optical electric torque. Thus, the threshold intensity of TR5/(5CB F-LC2) and TR5/(5CB F-LC4) became lower than that of TR5/(5CB F-LC1) and TR5/(5CB F-LC3).

In addition to the optical-electric torque, the elastic torque of LCs contributes to the sensitivity of the OFT. The elastic torque is proportional to a Frank elastic constant, *K*_ii_ [11]. In particular, the *K*_33_ elastic constant of LCs has a large effect on the OFT threshold in a homeotropic cell. Table 2 shows the *K*_33_ elastic constant of each sample. The values of *K*_33_ elastic constant of TR5/5CB, TR5/(5CB F-LC1), TR5/(5CB F-LC2), TR5/(5CB F-LC3), and TR5/(5CB F-LC4) were 9.1, 11.1, 3.1, 10.1, and 6.0, respectively. This result indicates that a lower *K*_33_ elastic constant enhances the sensitivity of the OFT.

Furthermore, we assume that the dye torque was changed by the substituents of host LCs. Marrucci reported that the intermolecular interaction between the anthraquinone dye and the host LC changes according to the substituents of the dye, leading to a change in the dye torque [10,24,25]. Although the mechanism of enhancing the OFT sensitivity in oligothiophene and anthraquinone systems may be different, intermolecular interactions between the oligothiophene dye and host LCs could vary depending on the terminal substituents of the host LCs, resulting in the dye torque change. This study focused on the optical-electric torque, the elastic torque, and the dye torque, while the effect of the optical-electric torque and the dye torque on the OFT sensitivity is not identified experimentally. Surface anchoring may also affect the OFT sensitivity. Detailed analysis is under investigation. The experimental identification of the mechanism will aid in the development of high-performance optical devices.

## 4. Conclusions

In conclusion, we investigated the OFT of oligothiophene-doped LCs using various host LCs with fluorine substituents. The molecular reorientation was induced by the irradiation with a linearly polarized laser beam. We found that the maximum number of rings of TR5/5CB, TR5/(5CB F-LC3), and TR5/(5CB F-LC4) with biphenyl structures was more than TR5/(5CB F-LC1) and TR5/(5CB F-LC2) with a phenyl ring. The difference in the conjugation length depending on the number of biphenyl rings affected the maximum number of rings. Furthermore, the threshold intensities of TR5/(5CB F-LC2) and TR5/(5CB F-LC4) became 42% lower than that of TR5/5CB. The reduction of the threshold can be explained by the changes in the optical-electric torque, the elastic torque, and the dye torque. The study revealed that the structure of the host LCs affected the sensitivity of the OFT. 

## Figures and Tables

**Table 1:** The TR5 concentration and absorption at 488 nm of the samples.

Sample	Concentration of TR5 (mol%)	Abs. at 488 nm
TR5/5CB	0.10	0.155
TR5/(5CB F-LC1)	0.13	0.155
TR5/(5CB F-LC2)	0.10	0.162
TR5/(5CB F-LC3)	0.12	0.161
TR5/(5CB F-LC4)	0.10	0.158

**Table 2:** Threshold intensity, the maximum number of rings, and *K*_33_ elastic constant for TR5/5CB, TR5/(5CB F-LC1), TR5/(5CB F-LC2), TR5/(5CB F-LC3), and TR5/(5CB F-LC4).

Sample	Threshold Intensity (W/cm^2^)	Maximum Number of Rings	*K*_33_ (pN)
TR5/5CB	20.1	23	9.1
TR5/(5CB F-LC1)	25.5	18	11.1
TR5/(5CB F-LC2)	12.0	18	3.1
TR5/(5CB F-LC3)	18.9	23	10.1
TR5/(5CB F-LC4)	11.7	22	6.0

## Data Availability

The authors confirm that the data supporting the findings of this study are available within the article.

## References

[1] Schadt M. (2009). Milestone in the history of field-effect liquid crystal displays and materials. Jpn. J. Appl. Phys..

[2] Lampert C.M. (2003). Large-area smart glass and integrated photovoltaics. Sol. Energy Mater. Sol. Cells.

[3] Priimagi A., Barrett C., Shishido A. (2014). Recent twists in photoactuation and photoalignment control. J. Mater. Chem. C.

[4] Tabiryan N.V., Sukhov A.V., Zel’Dovich B.Y. (1986). Orientational optical nonlinearity of liquid crystals. Mol. Cryst. Liq. Cryst..

[5] Simoni F., Bartolino R. (1985). Nonlinear Optical Behavior of Hybrid Aligned Nematic Liquid Crystals. Opt. Commun..

[6] Zel’dovich B.Y., Pilipetskii N.F., Sukhov A.V., Tabiryan N.V. (1980). Giant optical nonlinearity in the mesophase of a nematic liquid crystal (NCL). JETP Lett..

[7] Zolot’ko A.S., Kitaeva V.F., Kroo N., Sobolev N.N., Chilag L. (1980). Finding of optical nonlinearity. JETP Lett..

[8] Durbin S.D., Arakelian S.M., Shen Y.R. (1981). Optical-field-induced birefringence and Freedericksz transition in a nematic liquid crystal. Phys. Rev. Lett..

[9] Janossy I., Lloyd A.D., Wherrett B.S. (1990). Anomalous Optical Freedericksz Transition in an Absorbing Liquid Crystal. Mol. Cryst. Liq. Cryst..

[10] Marrucci L. (2002). Mechanisms of giant optical nonlinearity in light-absorbing liquid crystals: A brief primer. Liq. Cryst. Today.

[11] Janossy I., Lloyd A.D. (1991). Low-Power Optical Reorientation in Dyed Nematics. Mol. Cryst. Liq. Cryst..

[12] Zhang H., Shiino S., Shishido A., Kanazawa A., Tsutsumi O., Shiono T., Ikeda T. (2000). A thiophene liquid crystal as a novel π-Conjugated dye for photo-manipulation of molecular alignment. Adv. Mater..

[13] Matsumoto K., Usui K., Akamatsu N., Shishido A. (2020). Molecular reorientation behavior of oligothiophene-doped polymer-stabilized liquid crystals irradiated with collimated laser beam. Mol. Cryst. Liq. Cryst..

[14] Usui K., Matsumoto K., Katayama E., Akamatsu N., Shishido A. (2021). A Deformable Low-Threshold Optical Limiter with Oligothiophene-Doped Liquid Crystals. ACS Appl. Mater. Interfaces.

[15] Usui K., Katayama E., Wang J., Hisano K., Akamatsu N., Shishido A. (2017). Effect of surface treatment on molecular reorientation of polymer-stabilized liquid crystals doped with oligothiophene. Polym. J..

[16] Wang J., Aihara Y., Kinoshita M., Mamiya J.I., Priimagi A., Shishido A. (2015). Laser-Pointer-Induced Self-Focusing Effect in Hybrid-Aligned Dye-Doped Liquid Crystals. Sci. Rep..

[17] Yaegashi M., Shishido A., Shiono T., Ikeda T. (2005). Effect of ester moieties in dye structures on photoinduced reorientation of dye-doped liquid crystals. Chem. Mater..

[18] Yaegashi M., Kinoshita M., Shishido A., Ikeda T. (2007). Direct Fabrication of Microlens Arrays with Polarization Selectivity. Adv. Mater..

[19] Aihara Y., Kinoshita M., Wang J., Mamiya J.I., Priimagi A., Shishido A. (2013). Polymer stabilization enhances the orientational optical nonlinearity of oligothiophene-doped nematic liquid crystals. Adv. Opt. Mater..

[20] Wang J., Aihara Y., Kinoshita M., Shishido A. (2015). Effect of polymer concentration on self-focusing effect in oligothiophene-doped polymer-stabilized liquid crystals. Opt. Mater. Exp..

[21] Durbin S.D., Arakelian S.M., Shen Y.R. (1981). Laser-induced diffraction rings from a nematic-liquid-crystal film. Opt. Lett..

[22] Hird M. (2007). Fluorinated liquid crystals—Properties and applications. Chem. Soc. Rev..

[23] Zhu S., Chen R., Zhang W., Niu X., Chen W., Mo L., Hu M., Zhang L., Li J., Chen X. (2020). Dissecting terminal fluorinated regulator of liquid crystals for fine-tuning intermolecular interaction and molecular configuration. J. Mol. Liq..

[24] Marrucci L., Paparo D., Maddalena P., Massera E., Prudnikova E., Santamato E. (1997). Role of guest-host intermolecular forces in photoinduced reorientation of dyed liquid crystals. J. Chem. Phys..

[25] Marrucci L., Paparo D., Vetrano M.R., Colicchio M., Santamato E., Viscardi G. (2000). Role of dye structure in photoinduced reorientation of dye-doped liquid crystals. J. Chem. Phys..

